# Access to Syringe Services Programs — Kentucky, North Carolina, and West Virginia, 2013–2017

**DOI:** 10.15585/mmwr.mm6718a5

**Published:** 2018-05-11

**Authors:** Danae Bixler, Greg Corby-Lee, Scott Proescholdbell, Tina Ramirez, Michael E. Kilkenny, Matt LaRocco, Robert Childs, Michael R. Brumage, Angela D. Settle, Eyasu H. Teshale, Alice Asher

**Affiliations:** ^1^Division of Viral Hepatitis, National Center for HIV/AIDS, Viral Hepatitis, STD, and TB Prevention, CDC; ^2^HIV/AIDS Branch, Division of Epidemiology, Kentucky Department for Public Health, Cabinet for Health & Family Services; ^3^Injury and Violence Prevention Branch, Chronic Disease and Injury Section, Division of Public Health, North Carolina Department of Health and Human Services; ^4^Kanawha-Charleston Health Department, Charleston, West Virginia; ^5^Cabell-Huntington Health Department, Huntington, West Virginia; ^6^Louisville Metro Syringe Exchange Program, Louisville Metro Public Health and Wellness, Louisville, Kentucky; ^7^North Carolina Harm Reduction Coalition, Raleigh, North Carolina; ^8^West Virginia Health Right, Inc., Charleston, West Virginia.

The Appalachian region of the United States is experiencing a large increase in hepatitis C virus (HCV) infections related to injection drug use (IDU) (*1*). Syringe services programs (SSPs) providing sufficient access to safe injection equipment can reduce hepatitis C transmission by 56%; combined SSPs and medication-assisted treatment can reduce transmission by 74% (*2*). However, access to SSPs has been limited in the United States, especially in rural areas and southern and midwestern states (*3*). This report describes the expansion of SSPs in Kentucky, North Carolina, and West Virginia during 2013–August 1, 2017. State-level data on the number of SSPs, client visits, and services offered were collected by each state through surveys of SSPs and aggregated in a standard format for this report. In 2013, one SSP operated in a free clinic in West Virginia, and SSPs were illegal in Kentucky and North Carolina; by August 2017, SSPs had been legalized in Kentucky and North Carolina, and 53 SSPs operated in the three states. In many cases, SSPs provide integrated services to address hepatitis and human immunodeficiency virus (HIV) infection, overdose, addiction, unintended pregnancy, neonatal abstinence syndrome, and other complications of IDU. Prioritizing development of SSPs with sufficient capacity, particularly in states with counties vulnerable to epidemics of hepatitis and HIV infection related to IDU, can expand access to care for populations at risk.

## Kentucky

Before new legislation* in March 2015, SSPs were illegal in Kentucky. The new law allowed public health departments to operate SSPs after approval from relevant county boards of health, county fiscal courts, and city councils. Extensive education of official and community members about SSPs, addressing of concerns, and provision of data to dispel misimpressions (e.g., concerns that SSPs enable drug use) were required to achieve multiple levels of approval. Some counties held town hall meetings, inviting community members to learn about SSPs and have their questions answered. Counties that went through this process before beginning SSP operations reported increased support from law enforcement, the judicial system, community leaders, and community members. By the end of 2015, three counties in Kentucky had operational SSPs (Figure), including in the two largest cities, Louisville and Lexington. By August 2017, 31 counties had operational SSPs serving an estimated 8,078 clients; five counties had full approval but were not yet operational; and 10 counties were in some stage of gaining approval. Among 54 counties considered vulnerable to outbreaks of HIV and HCV (*4*), 21 (39%) had SSPs that were operational or approved to open by August 1, 2017. Location within public health departments facilitates client access to many other services (Table). Ten local health departments have their SSP integrated into daily public health clinics, so they are open 4 or 5 days per week, averaging 7.5 hours per day.

**FIGURE:**
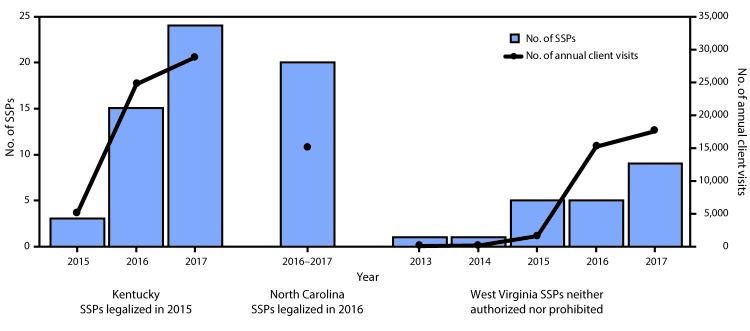
Syringe service programs (SSPs) and client visits to SSPs by persons who inject drugs — Kentucky, North Carolina, and West Virginia, 2013–2017 * Current as of August 1, 2017. ^†^ North Carolina’s visits represent total attendees for the first full year of operation. Kentucky and West Virginia reported data on a calendar-year basis.

**TABLE:** Services offered by syringe service programs — Kentucky, North Carolina, and West Virginia, as of August 1, 2017

Services	Kentucky, no. (%)	North Carolina, no. (%)	West Virginia, no. (%)
**Needle and syringe exchange**	24 (100)	20 (100)	9 (100)
**Other drug paraphernalia provided**			
Filters	14 (58)	—	6 (67)
Cookers	11 (46)	—	6 (67)
Sterile water	8 (33)	—	6 (67)
Alcohol wipes or swabs	21 (88)	—	7 (78)
Tourniquets	14 (58)	—	5 (56)
**Service delivery models**			
Fixed site	24 (100)	16 (80)	9 (100)
Peer counselors or peer workers	8 (33)	13 (65)	6 (67)
Mobile services	2 (8)	13 (65)	5 (56)
Secondary or peer-delivery model	1 (4)	0 (0)	5 (56)
Delivery	0 (0)	0 (0)	1 (11)
Pharmacy distribution	0 (0)	0 (0)	1 (11)
**Education provided**			
Safe injection practices	23 (96)	20 (100)	7 (78)
Naloxone administration	17 (71)	20 (100)	8 (89)
**Wound care**	17 (71)	—	7 (78)
**Hepatitis B**			
Vaccination	13 (54)	—	7 (78)
Screening	6 (25)	—	7 (78)
Linkage to treatment	22 (92)	—	7 (78)
**Hepatitis C**			
Screening	20 (83)	8 (40)	7 (78)
Linkage to treatment	24 (100)	20 (100)	9 (100)
**Human immunodeficiency virus**			
Screening	20 (83)	11 (55)	9 (100)
Linkage to treatment	24 (100)	18 (90)	6 (67)
Contact tracing and partner services	6 (25)	—	5 (56)
**Sexually transmitted diseases**			
Condom provision	24 (100)	—	9 (100)
Screening	16 (67)	—	9 (100)
Treatment	13 (54)	—	8 (89)
**Substance use disorder**			
Motivational interviewing	13 (54)	—	4 (44)
Linkage to medication assisted treatment	24 (100)	—	5 (56)
Linkage to behavioral treatment	24 (100)	17 (85)	6 (67)
**Reproductive health**			
Family planning services	14 (58)	—	8 (89)
Pregnancy testing	15 (63)	—	9 (100)
Linkage to prenatal services	20 (83)	—	8 (89)
**Social services**			
Housing assistance	6 (25)	—	3 (33)
Transportation assistance	6 (25)	—	3 (33)
Food assistance	6 (25)	—	3 (33)
Health insurance enrollment	10 (42)	—	3 (33)
**Mean (median [range]) hrs per week**	12 (3 [1.5–42.5])	18 (8 [4–60])	10 (4 [2–50])

## North Carolina

In 2013, the North Carolina legislature passed the 911 Good Samaritan/Naloxone Access Law^†^ and a law protecting persons from being charged for possession of drug paraphernalia if they alert a law enforcement officer to the presence of a hypodermic needle or other sharp object before search by the officer. On July 11, 2016, new legislation allowed any governmental or nongovernmental organization that “promotes scientifically proven ways of mitigating health risks associated with drug use” to start an SSP. Organizations were required to notify the North Carolina Safer Syringe Initiative (NCSSI) in the North Carolina Division of Public Health of the intention to establish an SSP before commencing operations. Registered programs are required to report data (e.g., services offered, referrals made, and syringes dispensed and returned) to NCSSI on an annual basis. As of August 1, 2017, 20 operational SSPs (Figure) served an estimated 3,983 clients in 52 of North Carolina’s 100 counties. SSPs are sponsored by 10 harm reduction coalitions, three churches or church partners of harm reduction coalitions, two acquired immunodeficiency syndrome (AIDS) service organizations, two local health departments, two substance use treatment centers, and a drug user union, offering services through a variety of models (Table). None of five counties in North Carolina classified as vulnerable to outbreaks of HIV and HCV (*4*) had SSPs during the first year of the program although some residents of vulnerable counties are served by existing SSPs.

## West Virginia

SSPs are neither prohibited nor expressly permitted by state law in West Virginia. The first known SSP began operation in a free clinic, fully integrated with primary health care services. In 2015, the West Virginia Bureau for Public Health funded a pilot project at the Cabell-Huntington Health Department as proposed by the Mayor’s Office of Drug Control Policy in Huntington, West Virginia. The West Virginia Harm Reduction Coalition was formed in February 2017 to support harm reduction activities and SSPs operating in the state. As of August 1, 2017, nine SSPs were known by the coalition to be operating in the state (Figure) serving an estimated 4,376 clients; four of these SSPs were located in three (11%) of 28 counties classified as vulnerable to outbreaks of HIV and HCV (*4*). Seven known SSPs were run by local health departments, and two operated out of free clinics, thereby facilitating access to other services needed by persons who inject drugs (Table). All SSPs were based in fixed sites; five also offered mobile services, five offered peer delivery (delivery of sterile injection equipment through a peer intermediary), and six had peer counselors (Table).

## Discussion

During 2013–2017, the number of operational SSPs increased from one to approximately 50 in Kentucky, North Carolina, and West Virginia. Visits to SSPs by clients who inject drugs also increased. In Kentucky and North Carolina, this increase followed changes in laws permitting access to sterile injecting supplies; in West Virginia, SSPs were never prohibited under state law. In North Carolina, any group can start an SSP after notifying the state health department; Kentucky requires a lengthy approval process for local health departments before offering syringe services. This paper demonstrates that increasing access to SSPs is possible with community support using a variety of models if SSPs are not prohibited by law.

The increase in client visits to SSPs by persons who inject drugs represents an unprecedented opportunity to improve access to care for this highly stigmatized population. In addition to increased access to sterile needles, syringes, and injection paraphernalia (*5*), comprehensive syringe services programs should also improve access to medication-assisted treatment, counseling, and social support to address substance use disorder (*6*); naloxone and lay naloxone training to prevent fatal overdose (*7*); the full range of contraceptives, including long acting reversible contraceptives to prevent unintended opioid-exposed pregnancy; prenatal care and medication-assisted treatment to reduce harm from substance use disorder in pregnant women and their infants (*8*); vaccination; and HCV, HIV, and hepatitis B virus (HBV) screening and treatment (*5*). State and local health departments that are actively addressing the health effects of the opioid crisis might consider a formal evaluation process to improve service quality and access for persons who inject drugs, including those attending SSPs. Process evaluation indicators for SSPs should include number of clients, number of syringes distributed, number of syringes returned, availability of services in hours per week, summary statistics on HIV, HBV, and HCV testing, and number and type of services (e.g., patient-centered family planning services and naloxone) and referrals provided (e.g., medication assisted treatment, prenatal care, HIV, and hepatitis treatment) (*9*). Evaluation should also include health indicators such as rates of hepatitis, HIV, fatal and nonfatal overdose, unintended pregnancy and neonatal abstinence syndrome, and initiation and retention in drug treatment. CDC has published a framework to guide evaluation of public health programs (*10*), which might be useful for evaluating access to essential services at the community level for persons who inject drugs.

The findings in this report are subject to at least six limitations. First, data were self-reported from SSPs and are therefore subject to bias. Second, because some programs do not collect identifying information, the total numbers of clients served is estimated. Third, at the time of this analysis, North Carolina was in its first year of implementation, and limited data are available. Fourth, no data were obtained for SSPs operating underground (i.e., outside the legal framework). Fifth, growth of SSPs and service integration in these states is rapid, and the most recent data on SSPs should be sought through the state or local health department or harm reduction coalition. Finally, these data cannot be used to evaluate quality of service delivery and whether service delivery is adequate to meet the needs of the population.

SSPs can be implemented through a variety of models and by a variety of agencies and organizations including those in rural areas. Demand for syringe services is growing rapidly in these three states with underserved populations of persons who inject drugs, representing an opportunity to implement, evaluate, and improve access to evidence-based services known to reduce the considerable morbidity and mortality associated with injection drug use.

**Summary**

**What is already known about this topic?**

Opioid overdose, human immunodeficiency virus, and viral hepatitis have increased among persons who inject drugs in the United States. Comprehensive syringe services programs (SSPs) reduce risks associated with injection drug use (IDU); however, access to SSPs has been limited.

**What is added by this report?**

SSPs have increased dramatically in Kentucky, North Carolina and West Virginia with support from government officials, community advocates, and healthcare providers.

**What are the implications for public health practice?**

Comprehensive SSPs can mitigate the health effects of IDU. With appropriate authorization and support, agencies can successfully implement SSPs in underserved areas.

## Footnotes

* https://law.justia.com/codes/kentucky/2015/chapter-218a/section-218a.500.

† https://www.ncleg.net/EnactedLegislation/Statutes/PDF/BySection/Chapter_90/GS_90-96.2.pdf.
