# *Erratum:* Vol. 67, No. 18

**DOI:** 10.15585/mmwr.mm6724a6

**Published:** 2018-06-22

In the report “Access to Syringe Services Programs — Kentucky, North Carolina, and West Virginia, 2013–2017,” on page 529, the second sentence should have read “**In combination with medication-assisted treatment**, syringe services programs (SSPs) providing sufficient access to safe injection equipment can reduce **the risk for hepatitis C acquisition by 74%** (2).”

